# Ion channel trafficking implications in heart failure

**DOI:** 10.3389/fcvm.2024.1351496

**Published:** 2024-02-14

**Authors:** Jean-Baptiste Reisqs, Yongxia Sarah Qu, Mohamed Boutjdir

**Affiliations:** ^1^ Cardiovascular Research Program, VA New York Harbor Healthcare System, New York, NY, United States; ^2^ Department of Cardiology, New York Presbyterian Brooklyn Methodist Hospital, New York, NY, United States; ^3^ Department of Medicine, Cell Biology and Pharmacology, State University of New York Downstate Health Sciences University, New York, NY, United States; ^4^ Department of Medicine, New York University Grossman School of Medicine, New York, NY, United States

**Keywords:** ion channels, trafficking, heart failure, electrophysiology, arrhythmia, treatment

## Abstract

Heart failure (HF) is recognized as an epidemic in the contemporary world, impacting around 1%–2% of the adult population and affecting around 6 million Americans. HF remains a major cause of mortality, morbidity, and poor quality of life. Several therapies are used to treat HF and improve the survival of patients; however, despite these substantial improvements in treating HF, the incidence of HF is increasing rapidly, posing a significant burden to human health. The total cost of care for HF is USD 69.8 billion in 2023, warranting a better understanding of the mechanisms involved in HF. Among the most serious manifestations associated with HF is arrhythmia due to the electrophysiological changes within the cardiomyocyte. Among these electrophysiological changes, disruptions in sodium and potassium currents’ function and trafficking, as well as calcium handling, all of which impact arrhythmia in HF. The mechanisms responsible for the trafficking, anchoring, organization, and recycling of ion channels at the plasma membrane seem to be significant contributors to ion channels dysfunction in HF. Variants, microtubule alterations, or disturbances of anchoring proteins lead to ion channel trafficking defects and the alteration of the cardiomyocyte's electrophysiology. Understanding the mechanisms of ion channels trafficking could provide new therapeutic approaches for the treatment of HF. This review provides an overview of the recent advances in ion channel trafficking in HF.

## 1 Clinical presentation

Heart failure (HF) is a chronic disease depicted by the inability of the myocardium to propel blood and oxygen effectively to comply with the body's needs and remains a major cause of death. It occurs when the heart muscles weaken or stiffen, leading to a reduced ability to pump blood. This cardiac pathology is characterized by dyspnea, peripheral edema, elevated jugular venous pressure, and tachycardia (1, 2). HF is a multifactorial disease mostly caused by various factors such as valvular diseases, coronary artery disease, endocardial or pericardial abnormalities, and high blood pressure (3, 4). In the US, HF affects 5.7 million individuals and is responsible for approximately 300,000 deaths per year (5). The incidence of HF is 5–10 per 1,000 individuals and the prevalence is 1%–2% annually (3). HF is classified based on the left ventricular ejection fraction (EF) into three EF categories: HF with reduced (HFrEF, ≤40%), mildly reduced (HFmrEF, 41%–49%), and preserved EF (HFpEF, ≥50%) (6). Given the scarce literature on ion channel trafficking in HFmrEF and HFpEF, the focus of this review is on HFrEF.

Pharmacotherapy is used for the treatment of patients with HFrEF and for improving their survival. This includes, β-blockers, angiotensin-converting enzyme inhibitors, angiotensin receptor blockers, and mineralocorticoid receptor antagonists (MRAs). Two new classes of drugs, angiotensin receptor-neprilysin inhibitor, sacubitril/valsartan (ENTRESTO®), and sodium-glucose cotransporter 2 (SGLT2) inhibitors are also used (7, 8). Adding MRAs, including spironolactone and eplerenone to the standard therapy in HF, reduced morbidity and mortality (9). Aldosterone, an end product of the upregulated renin-angiotensin-aldosterone system in HF, has been implicated as playing a major role in the progression of HF (10). In addition to the promotion of inflammation and fibrosis, aldosterone has been responsible for electrical remodeling (11). The chronotropic action of aldosterone was averted by spironolactone, indicating the role of MRA in this response (12, 13). HF is also associated with increased activity of the sympathetic nervous system (14). This chronic elevated plasma catecholamines lead to desensitization of the β-adrenergic signaling pathway. This includes downregulation of the number of receptor proteins from enhanced degradation and loss of receptor function (15, 16). Increased sympathetic activity leads to the upregulation of β-adrenergic receptor kinase, which phosphorylates the β-adrenergic and uncouples it from the second messenger triggered downstream pathway (17, 18). The result is a modification in the signaling pathway from a mainly β1-receptor in the healthy myocardium to a combined β1/β2 receptors in HF. These changes lead to a reduction of 50%–60% in β-adrenergic signaling capacity in advanced HF (19). β-blockers are efficient in restoring the adrenergic signaling pathway. Despite substantial advances in treating HF, the incidence of HF continues to rapidly increase posing a significant burden on human health. The total cost of healthcare for HF is projected to be USD 69.8 billion in 2023 (20).

Changes in the functional activity of ion channels play an important role in HF (21). Nevertheless, the mechanisms responsible for these alterations remain not fully understood. This incomplete knowledge can be attributed to the complexity of ion channels trafficking, which involves several proteins and serves as a significant contributor to the arrhythmogenic process. This review presents an update on the current understanding of ion channels trafficking physiology and, subsequently, provides the evidence related to trafficking defects observed in HF.

## 2 Electrical remodeling

### 2.1 Action potential

**Figure 1:**
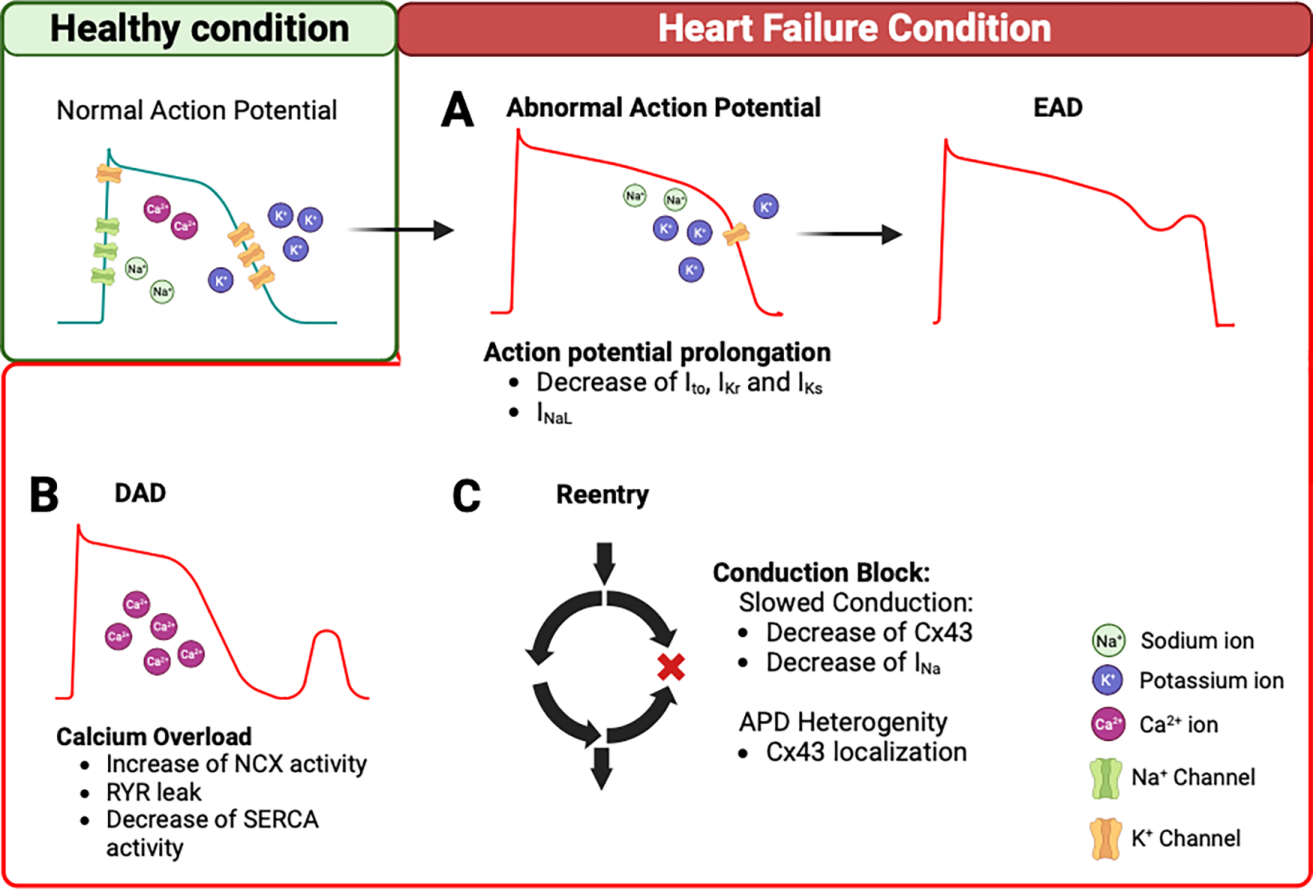
Arrhythmogenesis mechanisms in heart failure. (**A**) In HF condition, decrease of K^+^ current (I_to_, I_Kr_, I_Ks_) and/or I_NaL_ can prolong the AP. This remodeling triggers EADs. (**B**) In addition, intracellular Ca^2+^ overload during the diastole can trigger DADs. This is due to an increase in NCX reverse mode, Ca^2+^ leak from the RYR2, and a decrease of SERCA function. (**C**) The remodeling of Cx43 and/or decrease of I_Na_ contribute to slowing the conduction velocity and APD heterogeneity, which creates conduction block and promotes re-entry pathways. AP, action potential; EADs, early aftredepolarizations; DADs, delayed aftredepolarizations; NCX, Na^+^/Ca^2+^ exchanger; RYR2, ryanodine receptor 2; SERCA, Sarcoendoplasmic Reticulum Calcium ATPase; Cx43, connexin 43; APD, action potential duration.

The cardiac action potential (AP) is important for the generation and the propagation of excitation leading to contraction and is the result of ion channels composition in each cardiomyocyte (22–24). AP corresponds to a transient depolarization due to the sequential activation and inactivation of voltage-dependent ion channels, ensuring a flow of ions between the intracellular and extracellular fluid according to the electrochemical gradient. AP is composed of five phases in the non-pacemaker myocytes that determine its duration and amplitude (Figure 1). It is carried by a sequential activation and inactivation of different currents (25, 26). The depolarization phase (Phase 0) is generated by the inward sodium (Na^+^) current (I_Na_) encoded by the Na^+^ voltage-gated channel alpha subunit 5 (*SCN5A*). This phase is followed by a rapid repolarization (Phase 1), carried by the transient outward potassium (K^+^) current (I_to_) encoded by the K^+^ voltage-gated channel subfamily D 2 (*KCND2*). This repolarization is very fast and followed by a plateau phase (Phase 2), which is a balance between the ion calcium (Ca^2+^) inflow through L-type Ca^2+^ current (I_CaL_), encoded by the Ca^2+^ voltage-gated channel subunit alpha 1C (*CACNA1C*) and the outward flow of K^+^ ions. The rapid (I_Kr_) and the slow (I_Ks_) K^+^ currents, conduct this outward flow of K^+^, and are encoded by human ether-a-go-go–related gene (*hERG*) and by the K^+^ voltage-gated subfamily Q member1 (*KCNQ1*), respectively. These two currents terminate the AP resulting in the final repolarization (Phase 3). Finally, Phase 4 corresponds to the resting state around −80 mV in non-pacemaker cells, mainly held by the inwardly rectifying K^+^ currents (I_K1_) encoded by the K^+^ inwardly rectifying channel subfamily J 12/14 and 4 (*KCNJ12/14/4*) (27, 28) and also to the diastolic depolarization in the pacemaker cells carried by the funny current (I_f_) encoded by the hyperpolarization activated cyclic nucleotide gated K^+^ channel 4 (*HCN4*) (29, 30). Under pathological conditions such as HF, these ion channels undergo a remodeling process impacting the AP shape and AP duration (APD), part of which is due to trafficking disorders (31, 32). The APD of both atrial and ventricular cells is prolonged in HFrEF and HFpEF (33). However, Ca^2+^ handling is not impaired in the same way in HFrEF and HFpEF. In HFrEF, the systolic Ca^2+^ is decreased and the diastolic Ca^2+^ is increased, because of a decrease of T-tubule density and an increase of Na^+^/Ca^2+^ exchanger (NCX) expression. In HFpEF, there is a high cytoplasmic Ca^2+^ concentration associated with an unchanged NCX expression and an increased density of T-tubules (34).

### 2.2 Sodium current

I_Na_ alterations have been implicated in HF. A HF study conducted on mice missing the expression of the Muscle Lim Protein (MLP) demonstrated a decreased of I_Na_ density by post-translational reduction (35, 36). This I_Na_ reduction decreased the electrical conduction, favored re-entry, and contributed to inefficient synchronized conduction. Furthermore, studies conducted on dog and human HF led to alterations in the inactivation of I_Na_ causing a late persistent current (37, 38). Most of I_Na_ is rapidly inactivated, but a small fraction remains and contributes to generate a late I_Na_ (I_NaL_), thus contributing to increase in the concentration of Na^+^ ions in the cardiomyocyte and consequently increasing the APD (39). However, the molecular mechanisms which explain this I_NaL_ in HF are still unclear. One study showed that the heterologous expression of Nav1.5 produced a late opening in gating modes (40). Furthermore, another explanation for this late current would be the contribution of the neuronal isoform Nav1.1. One study, on a dog model of HF, demonstrated the participation of this neuronal isoform in inducing the I_NaL_ (41). Targeting the neuronal type of Na^+^ channel by riluzole was reported to reduce this I_NaL_ (42). Riluzole could thus offer a therapeutic target to prevent arrhythmia and slow the progression of HF (43). The importance of this I_NaL_ abnormality has been well demonstrated to contribute to arrhythmias in HF, and medications targeting the I_Na_ could prevent arrhythmia (44). In fact, some studies used ranolazine, an I_NaL_ inhibitor, to reduce and prevent arrhythmias in HF (45, 46). To this end, a medication empagliflozin, an SGLT2 inhibitor, was described to interact directly with the Na^+^ channel and reduce the I_NaL_ in a mouse model (47). Recently, another study used empagliflozin, a SGLT2 inhibitor, to revert the I_NaL_ upregulation and arrhythmogenic AP in an HFpEF mice model, induced by high-fat diet and nitric oxide synthases inhibition (48). These studies showed that inhibiting I_NaL_ may be a therapeutic target in the prevention of arrhythmia onset in HF (49). The accumulation of Na^+^ concentration can alter the Ca^2+^ homeostasis because the Ca^2+^ efflux is via the NCX, which will compensate by extruding excessive intracellular Na^+^ resulting in intracellular Ca^2+^ overload and subsequent triggered arrhythmic events (50).

### 2.3 Potassium current

In HF, alterations in I_Kr_, I_Ks_, and I_to_ can be present. I_K_ plays a crucial role in the cardiac AP shape, and its remodeling is an important contributor to repolarization abnormalities in HF (51, 52). First, the I_to_ is known to be reduced in HF and to participate in APD prolongation (53, 54). The reduction of I_to_ participates in increasing the Ca^2+^ influx and Ca^2+^ concentration in the cells, which triggered the activation of hypertrophic pathway in the mice model (55, 56). Hypertrophy is a compensatory response to HF, but sustained cardiac hypertrophy contributes to the development of HF. Remodeling in I_Kr_ and I_Ks_ in HF have also been reported (57–59). A study conducted in rabbits with atrioventricular block showed a reduction of both I_Kr_ and I_Ks_, using the patch-clamp technique (60, 61). I_K1_ was also shown to be downregulated in HF (59). The small-conductance Ca^2+^-activated K^+^ currents (SK) are upregulated in HF (62). This current participates in the repolarization of healthy atria but not in the ventricle (63). Other studies have shown an upregulation in this channel in ventricular myocytes, which prolongs the APD and promotes the arrhythmogenesis in HF (64, 65). Finally, a downregulation of the TREK channel was observed in patients with atrial fibrillation complicated by HF (66). In all, the remodeling of I_K_ in HF underlies, in part, the electrical abnormality and associated arrhythmogenesis (67).

### 2.4 Calcium handling

During the development of HF, important changes in Ca^2+^ handling occur (68). Key players in Ca^2+^ homeostasis include L-type Ca^2+^ channels, NCX, ryanodine receptor type 2 (RYR2), sarcoplasmic reticulum Ca^2+^-ATPase (SERCA), and Na^+^/H^+^ exchangers (NHE).

#### 2.4.1 Calcium current

Dysregulation of I_CaL_ can impair the ability of the heart to contract affectively, leading to a decreased contractility and pumping function. The alteration of Ca^2+^ channels, which allow Ca^2+^ entry in failing cardiomyocytes, is still debatable in HF. In fact, the existing data on Ca^2+^ channel gene expression during HF show either a decrease or small changes in transcript levels of the gene *CACNA1C* (69–71). In addition, the increased phosphorylation of the Ca^2+^ channel leads to an increase of its open probability in human failing ventricle, suggesting a compensatory mechanism (72, 73). Furthermore, our group has demonstrated the re-expression of alpha_1D_ L-type Ca^2+^ channels encoded by the CACNA1D gene in failing human ventricles, which is normally not expressed in the healthy ventricles (74). Their unique activation at a more negative voltage allows to play a larger role in Phase 4 depolarization and spontaneous beating. Furthermore, the density of I_CaL_ is influenced by the severity of failure (75). In HF, adult ventricular myocytes reactivate the fetal gene expression program and acquire spontaneous beating (76, 77). In addition, some studies reported the re-expression of T-type Ca^2+^ channels in post-infarcted hearts, in hypertrophied adult cat hearts and in rat models of HF (78–80). Despite these modifications, the L-type Ca^2+^ channels are the main sources of extracellular Ca^2+^.

#### 2.4.2 Sodium-calcium exchanger

NCX works in two modes: forward mode (Ca^2+^ efflux and Na^+^ influx) to reduce diastolic Ca^2+^ levels and reverse mode (Ca^2+^ influx/Na^+^ efflux) depends on the sarcolemma Na^+^ gradient (81). In HF, with an aberrant accumulation of Na^+^, NCX changes from forward to reversed mode, resulting in an increase of Ca^2+^ concentration in the cell (82, 83). The implication of NCX dysregulation has been demonstrated in human HF for triggering arrhythmias (84, 85). Studies have also demonstrated an upregulation of both mRNA and protein expression of NCX in human and animal models, contributing to the arrhythmogenicity of HF (86). These changes are associated with arrhythmogenic mechanisms, such as early (EADs) and delayed afterdepolarizations (DADs) in HF (87, 88). Therefore, the block of NCX may represent a potential therapy for the prevention of arrhythmia in HF (85). The selective NCX blocker, SEA-0400, has already demonstrated a potential benefit in pig and mice models of HF (89, 90). This NCX blocker restores the sarcoplasmic reticulum (SR) Ca^2+^ load and releases it, improving Ca^2+^ handling (91). Recently, new NCX blockers were developed, ORM-11035 and SAR296968, which have significantly attenuated Ca^2+^ handling remodeling and diastolic dysfunction in a rat model of HF (85, 92).

#### 2.4.3 Ryanodine receptor

Dysregulation of Ca^2+^ homeostasis can impair the capacity of the myocardium to contract efficiently, leading to a decreased contractility and pumping function. These modifications in Ca^2+^ handling result in higher diastolic Ca^2+^ and enhanced diastolic Ca^2+^ loss from the SR, together impacting the Ca^2+^ induced Ca^2+^ release mechanism and excitation–contraction coupling culminating in reduced contractile force. The RYR2 is a Ca^2+^-release channel primarily found in the SR of cardiomyocytes. Its function is to regulate the release of Ca^2+^ during the cardiac contraction. RYR2 is directly or indirectly linked to the pathological cellular mechanisms of HF (93, 94). It interacts with a multitude of proteins regulating its activity, such as protein kinase A (PKA) and calmodulin-dependent kinase II (CamKII) (95). When one of these elements is perturbed, the RYR2 undergoes a pathological remodeling, which can initiate or amplify the cellular mechanisms contributing to HF. PKA hyperphosphorylation of RYR2 in HF results in the depletion of its stabilizing FK506 binding protein, FKBP12.6 (96). RYR2 are hyperphosphorylated by PKA and/or CAMKII, causing aberrant Ca^2+^ leaks from the SR (97). The diastolic releases of SR Ca^2+^ have been linked to DADs triggering fatal ventricular arrhythmias (Figure 1). In a canine model of rapid pacing-induced HF, administration of a β-adrenergic blocker, metoprolol, reverses PKA hyperphosphorylation of RYR2, restoring the stoichiometry of RYR2 macrocomplex, and normalizes single-channel function (98–100). Recently it has been demonstrated that Entresto® diminished diastolic Ca^2+^ spark frequency and SR leaks in mouse myocytes subjected to catecholaminergic stress, as well as in human left ventricular cardiomyocytes with end stage HF (101). The findings suggest that Entresto® ameliorates cardiac function in part by improving myocardial Ca^2+^ homeostasis.

The impairment of SR function has proven to be predominantly induced by a SR Ca^2+^ load reduction due to decreased activity of the SERCA and also from the RYR2 leak (102–104). It is associated with a Ca^2+^ leak from the SR through the RYR2 and a higher RYR2 activity (105, 106). Other studies have highlighted that the SR Ca^2+^ leak triggers mitochondrial dysfunction and leads to an increased production of free radicals, which in turn leads to pathological RYR2 remodeling (107). A further SR Ca^2+^ leak through RYR2 is then observed with HF progression exacerbation (108). Further results published show that silencing the expression of junctophilin-2 in the mice provokes SR Ca^2+^ leak through RYR2, and the development of HF (109). Conversely, a study demonstrated that maintaining the level of junctophilin-2 protein at a high level can prevent the progression of HF (110). This study underlies a key role of junctophilin-2 in HF and could be an interesting therapeutic target for HF. One of the largest gene transfer clinical studies (CUPID2), targeting the SERCA activity, was initiated in patients with HFrEF (111). However, the preliminary results did not show any improvement of the heart function.

Collectively, the Ca^2+^ mishandling observed in HF contributes to reduced contractility of the myocardium triggering pump failure or arrhythmias. The Ca^2+^ cycling disruption is one of the central elements initiating and progressing the cardiac function deterioration in HF (72).

#### 2.4.4 Sodium/proton exchanger

The NHE simultaneously transports 1Na^+^ ion into the cells and extrudes 1H^+^ ion (112). NHE type 1 is ubiquitous in all tissue plasma membranes, including the heart (113). In cardiac cells, NHE is localized at intercalated disks and T-tubules, and may influence local pH (114). NHE activity is significantly increased in both patients with HF and animal models of HF (82, 115). The subsequent rise in intracellular Na^+^ stimulates the NCX and results in an increase in intracellular Ca^2+^, which promotes myocardial damage. The mechanism of SGLT2 inhibitor-mediated cardioprotection in HF is possibly through its effect on NHE. Specifically, SGLT2 inhibitors (empagliflozin, dapagliflozin, and canagliflozin) act directly on NHE, by cross reacting with the Na^+^-binding site of NHE, inhibiting the activity of NHE, thereby lowering the intracellular Na^+^ and thus producing cardioprotective effects (116). It was suggested that diminished NHE activity in the heart by SGLT2 inhibitors is due to the reduction in reactive oxygen species (ROS), oxidative stress production, cardiac inflammation, and fibrosis (117). Altogether, inhibition of NHE could open a new therapeutic avenue for HF (115).

### 2.5 Pacemaker current

Clinical and experimental studies have demonstrated that the sinus node function is impaired in HF (118, 119). These observations are correlated with a downregulation of the I_f_ in the sinus node pacemaker in a rabbit HF model (120). Another study in dogs with HF reported a decrease of the transcript and protein level of both *HCN4* and *HCN2*, which explains the decrease of I_f_ and the impairment of the sinus node function indicating that the pacemaker current is remodeled in HF (119). Unlike these studies, the study conducted on human HF demonstrated an upregulation of both atrial and ventricular *HCN2* and *HCN4*, accounts for the observed increase in I_f,_  which could contribute to atrial and ventricular arrhythmias (121). A healthy adult ventricle normally has low levels of *HCN2* and *HCN4.* However, the expressions of these genes increases as a result of HF (122). In support of this study, clinical trials have demonstrated the beneficial effects of ivabradine, an I_f_ inhibitor, in HF (123–125). Additional studies are warranted to establish the exact role of I_f_ and its genes *HCN2/HCN4* in HF.

### 2.6 Connexins

The electrical propagation in cardiomyocytes occurs through the connexins and forms an electrical cell-to-cell coupling (126). The connexin-43 (Cx43) protein is highly expressed in the ventricles (127). In diseased hearts such as in the case of hypertrophy and HF, Cx43 is localized at the lateral side of the ventricular myocytes (128). While Cx43 remodeling has been associated with spontaneous ventricular arrhythmia, the exact mechanisms for arrhythmogenesis are just emerging. Interestingly, the expression of Cx43 has been described to be downregulated in HF condition (129, 130). In addition, a decreased phosphorylation of Cx43 was detected in HF cardiomyocytes, which impairs the function and the localization from the intercalated disc to the lateral membrane (131, 132). Abnormalities in function and expression of Cx43 result in slow conduction in HF, which increases the propensity for arrhythmic events. Lahnwong et al. showed that pretreatment with dapagliflozin, a SGLT2 inhibitor, in a rat model of HF had lower arrhythmia score (133). The anti-arrhythmic effects of SGLT2 inhibitor are likely explained by its effect on the phosphorylation level of Cx43.

These ion channels, exchangers, and connexins remodeling in HF increase the propensity of arrhythmias. EADs and DADs represent triggers of arrhythmias. EADs are due to AP prolongation caused by a reduction of repolarizing currents, such as I_to_, I_Kr_, and I_Ks_, and an increase the I_CaL_ and I_NaL_ (Figure 1A). The DADs are caused by Ca^2+^ handling abnormalities during the diastole, due to an increased activity of NCX, decreased SERCA function, and Ca^2+^ leak from the RYR (Figure 1B). Furthermore, the re-entry mechanisms are the result of the remodeling process that leads to slower conduction velocities and the heterogeneity of APD, which can create conduction blocks. A reduction, observed in the HF model, of I_Na_, I_f_, and Cx43 favors this phenomenon and contributes to sustained arrhythmic events (Figure 1C). These ion channel changes are summarized in Table 1.

**Table 1:** Ion channels involvement and heart failure arrhythmogenesis.

Ion channel	Status	Consequence	Mechanisms of arrhythmia	References
Na^+^ channel	• Reduced current • Abnormalities of inactivation	• Slowed cardiac conduction • Persistent I_Na_ • Prolonged AP	• Re-entry • EAD	(36, 134–136)
Ca^2+^ handling	• Increase of NCX reversed mode • Decrease of I_CaL_ • Increase of I_CaL_ phosphorylation • Decrease of SERCA function • Ca^2+^ leak from RYR	• Increase of Ca^2+^ entrance • Ca^2+^ overload • Impaired Ca^2+^-induced Ca^2+^-release	• DAD • Reduced contractility	(69, 74, 108, 117, 137)
K^+^ channel	• Decrease of I_to_, I_Kr_ and I_Ks_	• Slowing of the repolarization • Prolonged AP	• EAD	(51, 57, 61)
Pacemaker current	• Downregulation of HCN expression	• Reduced pacemaking function	• Bradycardia • Sino-atrial bloc	(120, 138)
Connexin	• Downregulation of Cx43 • Decrease of phosphorylation • Lateral localization	• Slowed conduction velocity • Increase in APD heterogeneity	• Re-entry	(130–132)

APD, action potential duration; Cx43, connexin–43; DAD, delayed afterdepolarization; EAD, early afterdepolarization; NCX, sodium/calcium exchanger; RYR, ryanodine receptor; ICa, calcium current; Ito, transient outward potassium current; IKr, rapid outward potassium current; IKs, slow outward potassium current.

## 3 Ion channel trafficking

The newly synthesized proteins go through the process of trafficking to reach their destination. The two major components are the endoplasmic reticulum (ER) and the Golgi apparatus and allow the synthesis of functional proteins. The ion channels trafficking into microdomains of the plasma membrane is critical for the anisotropic transmission of the electromechanical signal at the tissue level (139).

**Figure 2:**
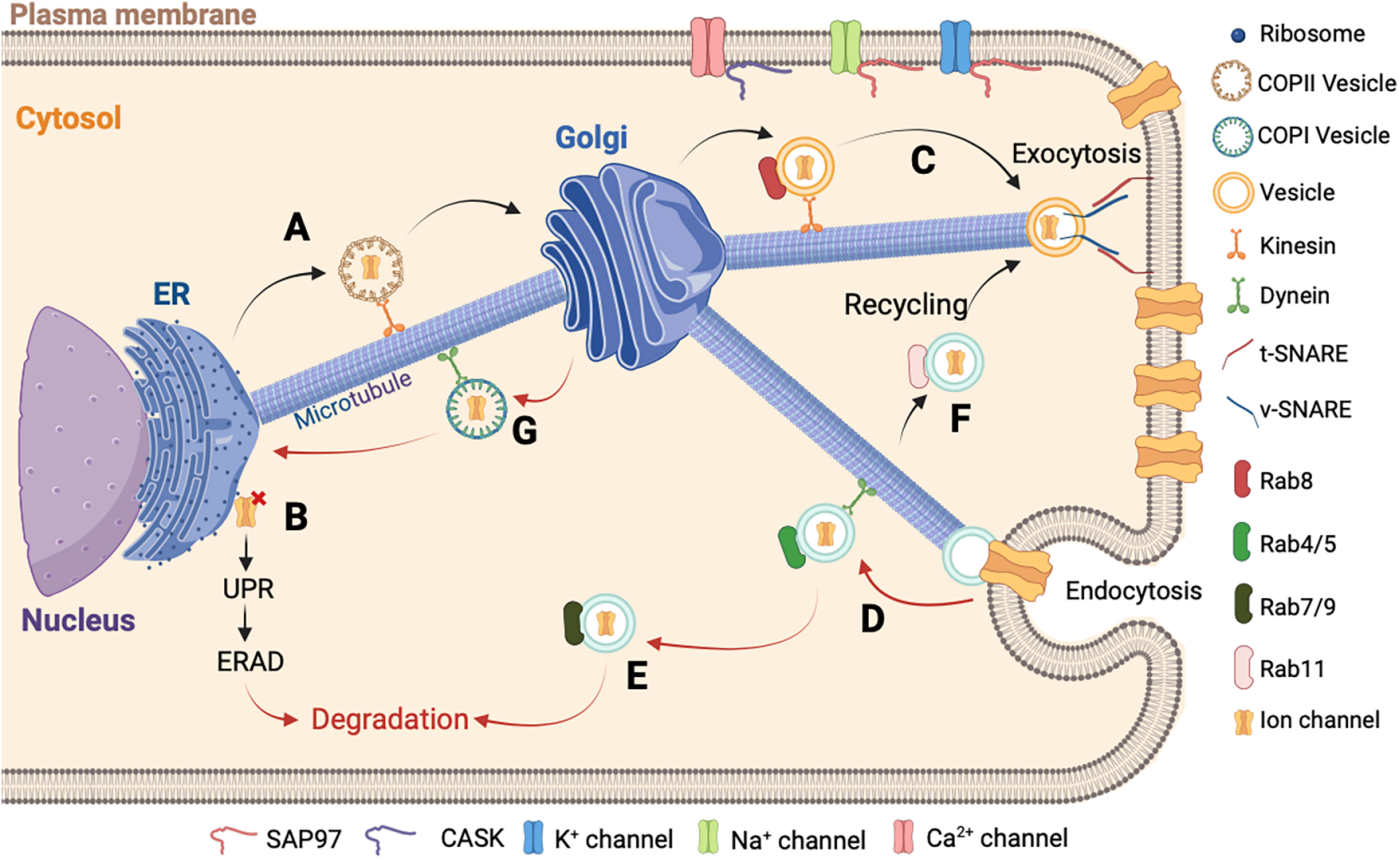
Schematic representation of ion channels trafficking. (**A**) The newly synthesized proteins in the ER are carried by the COPII vesicles until the Golgi apparatus in the anterograde direction. (**B**) In cases where the assembly of the newly synthesized protein is misfolded, it is retained in the ER followed by the activation of the UPR and ERAD to allow its degradation in the cytosol. (**C**) After the final maturation process, the new ion channel protein is directed to the plasma membrane by a vesicle. At the final localization, the v-SNARE and t-SNARE proteins interact to allow the fusion between the vesicle and the plasma membrane. (**D**) Then the ion channel is recaptured into the cell by endocytosis and carried out by a vesicle with Rab4/5 protein and dynein to allow the retrograde direction. (**E**) The ion channel is transported by a late vesicle with Rab7/9 protein and degraded in the cytosol, or (**F**) is recycled by another vesicle with the Rab11 protein. (**G**) The final step of the ion channel trafficking is carried out by the COPI vesicle for the retrograde direction to be recycled into the ER. It is important to note that the accessory molecules, such as CASK and SAP97, stabilize the ion channel at the destination.

The starting point of ion channels trafficking is in the ER, where the ion channels are synthetized by the ribosomes. A quality control is carried out in the ER before the protein is transported to the Golgi (140). The newly formed proteins are supported by chaperone proteins (calnexin, calreticulin) which will facilitate their maturation and their oligomerization (Figure 2A) (141). However, misassembled ion channels can exhibit the ER-retention signals. Among these signals, dual bi-arginine (RXR) and C-terminal Lys-Asp-Glu-Leu (KDEL) motifs are well known (142, 143). A misfolded protein in the ER triggers the unfolded protein response (UPR) using three pathways such as inositol-requiring enzyme 1 (IRE1), activating transcription factor 6 (ATF6), and protein kinase R-like ER kinase (PERK) (11, 144). These pathways will activate the genes involved in the ER-associated degradation system (ERAD) and induce a cytosolic degradation by the proteasome (Figure 2B) (145). In the Golgi, the ion channels undergo post-translational modifications, such as the glycosylation before being trafficked to the plasma membrane (146, 147). Many ion channels and their subunits are glycosylated. N-glycosylation will regulate the stability and the expression level of the membrane channels, their transport, and also their biophysical properties (148, 149).

Proteins will leave the ER through a region without ribosomes called ER Exist Site (ERES) (150, 151). In this region, the ion channels are packaged in coatomer protein II (COPII) vesicles. These COPII vesicles ensure anterograde transport from the ER to the Golgi, whereas the coatomer protein I (COPI) vesicles mediate the retrograde transport (Figure 2) (152, 153).

In cardiomyocytes, the transport of ion channels needs molecular motors and regulators. In the cytoplasm, the ion channel trafficking is coordinated by various Rab GTPases (154). Rab proteins belong to the largest family of small Ras-like GTPases. Active in the presence of GTP and inactive in the presence of GDP, they are associated with practically all stages of vesicular transport, including the Golgi stages. Rab8 is involved in the anterograde direction for the delivery of newly synthetized ion channels (155). Rab4 and Rab5 are associated with vesicles following ion channel internalization (156, 157). Rab7 and Rab9 are associated with the late endosome and Rab11 is associated with the recycling endosome (156, 158). In the anterograde direction, the transportation is provided by the action of different kinesins and thereafter along the actin cytoskeleton by the action of myosin V. In the retrograde direction, the dynein associated with the microtubules and actin carry out the transportation (Figure 2) (159, 160).

Another important aspect of ion channels regulation is their anchoring and aggregation in different areas of the sarcolemma. Many adapter proteins capable of interacting with the channels and regulating their membrane localizations have been identified, such as the membrane-associated guanylate kinase (MAGUK) protein family (161). Most MAGUKs have one or more PDZ domains, an SH3 domain, and a GUK domain. The presence of these many protein interaction domains allows MAGUK to interact with multiple partners simultaneously and explains their multiple roles such as anchoring and aggregation of ion channels in specialized areas of cellular communication, scaffolding of macromolecular complexes, functional interaction of channels with signaling elements, and intracellular trafficking (162). For example, cardiomyocytes express several MAGUK proteins, where the most important is SAP97 (synapse-associated protein 97), which interacts with the major ion currents, I_Kr_, I_K1_, and I_Na_ (Figure 2) (163–165). Another protein of the MAGUK family participates in the trafficking and anchoring of ion channels, the Ca^2+^/calmodulin-dependent serine protein kinase (CASK) protein. This proteins has a domain homologous to CAMKII. The CASK protein is composed of the combination of typical MAGUK domains plus two L27 domains and an N-terminal CAMKII domain (166). Studies have demonstrated that the CASK protein is involved in the Ca^2+^ channel trafficking and its stabilization at the membrane level (Figure 2) (167). In addition, the expression and phosphorylation levels of *hERG* are dependent on the presence of CASK protein (168). Furthermore, CASK protein interacts with the Nav1.5 channel and regulates its trafficking and its surface expression at the lateral membrane (169).

The end stage of the ion channel trafficking corresponds to the attachment of vesicles to the membrane. This attachment is carried out by the N-ethylmaleimide sensitive fusion protein (NSF), the NSF attachment proteins (SNAP), and the SNAP REceptor (SNARE) proteins (170, 171). Each transport vesicle contains one or more v-SNAREs (v for vesicle), which interacts with a t-SNARE (t for target) on the target membrane. The assembly of the pair of SNARE proteins is regulated by different types of proteins including the GTPases of the Ypt/Rab family and the Sec1 family (170, 172). This process is catalyzed by NSF and SNAPs. This assembly will destabilize the lipid bilayers and permits the ion channel to be directed to the plasma membrane (Figure 2C) (173). Another contributor to the ion channel trafficking is ankyrin. Ankyrin constitutes a group of intracellular proteins responsible for arranging, transporting, and securing membrane protein complexes to the actin/spectrin cytoskeleton, thereby establishing microdomains within membranes that exhibit functional properties (174). The isoform G and B are present in the heart tissue and are implicated in the stabilization of ion channels at the membrane level (175). The ankyrin-G was identified to stabilize the Nav1.5 channel in the heart and is important for the localization at the intercalated discs as described in the ankyrin-G knock-out mice (176). The ankyrin-B interacts with the NCX, the Cav1.3 channel, and stabilizes the SERCA, three important Ca^2+^ transporters (177, 178). A defect of ankyrin-B was previously described to induce Ca^2+^ abnormalities, increase injury, and reduce contractility in a mouse heart that is ankyrin-B deficient (179).

It is important to note that the level of ion channels in the plasma membrane is also dependent on a balance between the phenomena of exocytosis and endocytosis, recycling, and degradation. This balance is important to allow a satisfactory regulation of ion channel activity.

## 4 Ion channel trafficking defect contributes to the development of heart failure

Cardiomyocytes have distinct microdomains, such as t-tubules, lateral membranes, and intercalated discs. These microdomains have precise physiological roles in ion channels trafficking to microdomains and hence it is crucial to maintain these specific roles. However, in HF, a mislocalization or redistribution of ion channels disrupts the normal electrical gradients and alters the propagation of electrical signals. The different aspects of the ion channel trafficking in HF are summarized in Table 2.

**Table 2:** Summary of ion channel trafficking alterations and their consequences in heart failure.

Trafficking alteration	Consequences	Ion channel implicated	References
Mutations	Degradation of ion channels	Na^+^, Ca^2+^, and K^+^ channel	(180–182)
Post-translational modification	Degradation of ion channels	Na^+^, Ca^2+^, and K^+^ channel	(183, 184)
ER stress	Activation of ERAD Ion channel degradation	Na^+^ and K^+^ channel	(185, 186)
Upregulation of detyrosination	Destabilization of microtubules	Na^+^ channel	(187, 188)
Alteration of EB1 protein Alteration of TIP protein	Destabilization of the anchor of the vesicles in the plasma membrane Reduction in the exocytosis	Na^+^ channel Cx43	(189–192)
Disturbance of BIN1 and CASK protein	Decrease in the role of scaffolding protein in the membrane Reduction in the exocytosis	Ca^2+^ channel	(193, 194)
Decrease of synapsin-2	Alteration in the association between the vesicle and the microtubule	NCX	(195)
HSP90	Alteration in the interaction between HSP and *hERG*	*hERG*	(196, 197)
Hypokalemia	Abnormal ubiquitination of *hERG*	*hERG*	(198)

BIN1, Myc box-dependent-interacting protein 1; CASK, calcium/calmodulin-dependent serine protein kinase; Cx43, connexin 43; EB1, plus-end binding protein; ER, endoplasmic reticulum; ERAD, endoplasmic reticulum associated protein degradation; HSP, heat shock protein NCX, sodium/calcium exchanger; TIP, microtubule plus-end tracking proteins.

**Figure 3:**
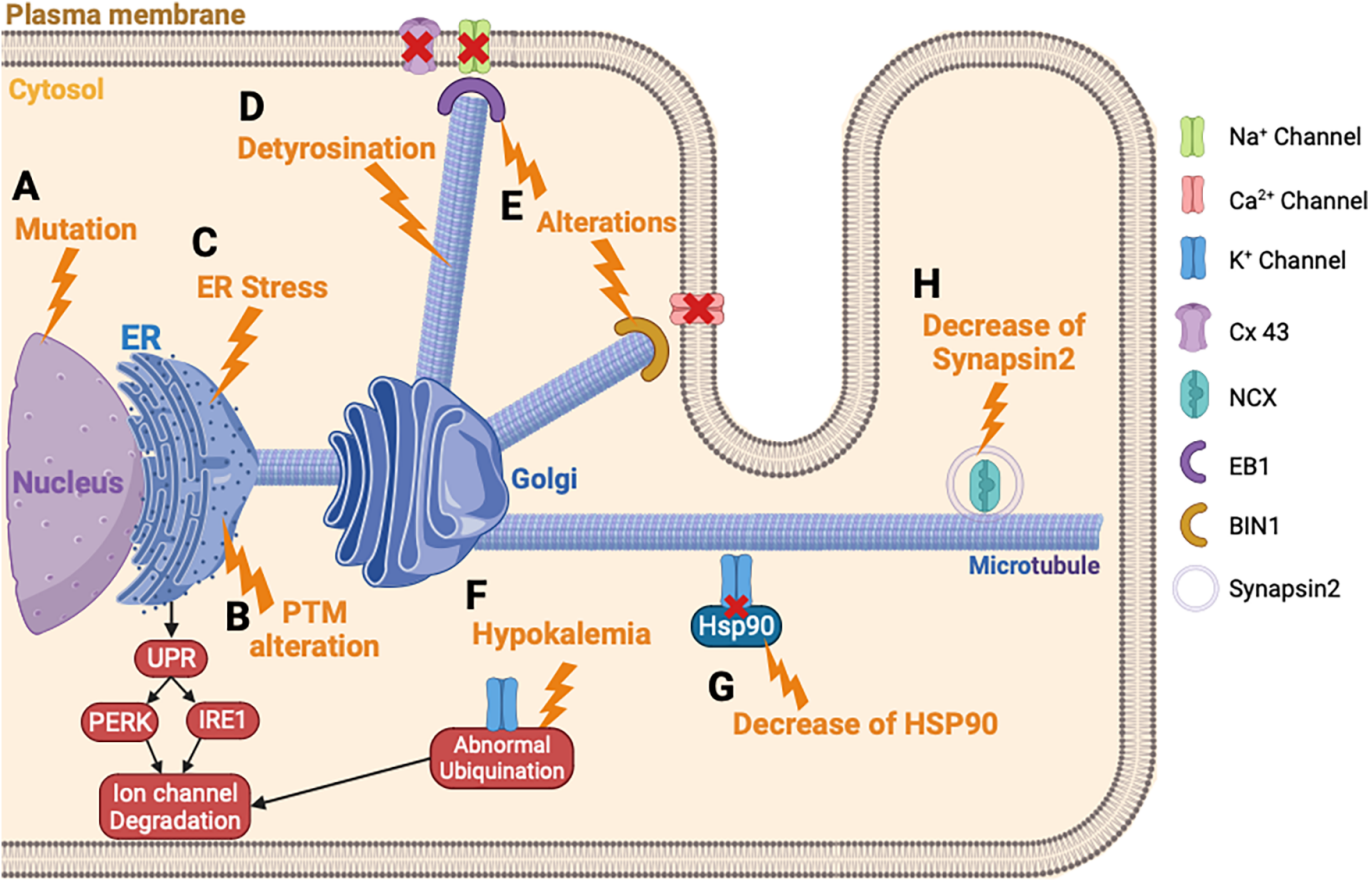
Ion channels trafficking perturbation in heart failure. (**A**) Ion channel variant leads to ER retention of the ion channel. (**B**) PTM alteration triggers the ion channel retention in the ER and the degradation of the ion channel. (**C**) ER stress leads to an abnormal degradation of the ion channel protein by the UPR response. (**D**) The detyrosination of the microtubules alters the ion channel trafficking. (**E**) Alteration of EB1 or BIN1 inhibits the ion channel incorporation into the membrane. (**F**) Hypokalemia triggers an abnormal ubiquitination of *hERG* and its degradation. (**G**) Decrease of HSP90 chaperone disturbs the K^+^ channel trafficking. (**H**) Decrease of synapsin2 vesicle decreases the trafficking of NCX. PTM, post-translational modification.

Since the 1990s, it was discovered that variants in ion channels are responsible for trafficking defects, leading to the development of HF and arrhythmias (Figure 3A) (199). Also, several abnormal post-translational modifications, which affect many cellular pathways, have been reported in patients with HF (183). Among these modifications, alterations in phosphorylation, glycosylation, ubiquitin, acetylation, and succinylation were reported (168, 200, 201). Several enzymes control these post-translational modifications and are summarized in Table 3. Alterations in these processes can lead to the degradation of channel proteins by the proteasome leading to electrical abnormalities such as the long QT syndrome or arrhythmias associated with HF, revealing its importance in the functional expression of ion channels (Figure 3B) (184). In recent years, the studies conducted on post-translational modifications have gradually increased and become a new field of medical research for the HF (207–209).

**Table 3:** Post-translational modifications in heart failure.

Modification	Enzyme	Modification site	Function	Reference
Phosphorylation	PKA, PKC CAMK MAPK	Serine Threonine Tyrosine	Protein synthesis and metabolism	(202)
Glycosylation	Glycosyltransferase	Serine Threonine Hydroxyproline	Protein folding and glycoprotein stability Protein sorting and packaging	(203)
Ubiquitin	E1, E2, E3	Lysine	Apoptosis Signal transduction	(204)
Acetylation	P300 GNAT family SRC family	Lysine	Metabolism signal transduction stress	(205)
Succinylation	GCN5 HAT1	Lysine	Inflammation	(206)

E, ubiquitin ligase; GCN5, general control non-depressible 5; GNAT, GCN5 related N-acetyltransferase; HAT1, histone acetyltransferase1; MAPK, mitogen-activated protein kinase; P300, histone acetyltransferase; SRC, proto-oncogene tyrosine-protein kinase.

The ER, which is the first step of the maturation and trafficking of ion channels, is impacted by HF, which induces a stress of this cellular compartment (210, 211). In mice injected with tunicamycin to induce HF, a prolonged activation of UPR and PERK is provoked, which activates the degradation pathway of the newly formed proteins by ERAD (Figure 3C) (185). This study showed that I_Na_, I_Kr_, and I_K1_ are downregulated by this mechanism, in response to ER stress induced by HF. Each ion channel has specific trafficking defects, detailed as follows.

### 4.1 Sodium current

Most trafficking defects come from a direct variant on ion channels, which induces a misfolded protein and the retention in ER. Variants in the Na^+^ channel were discovered to be linked with a trafficking defect, leading to HF (212). A recent study demonstrated that two different variants of Nav1.5 can interact with each other to rescue the I_Na_ (181). Specifically, this study showed that one variant of Na^+^ channel, trafficking-deficient but gating-competent, was able to restore a fraction of the I_Na_ by interacting with another Na^+^ channel variant that was trafficking-competent but gating-deficient. This study supports the notion that Na^+^ channel α-subunits interact and cooperate with each other. The interaction between Na^+^ channels will allow to rescue the trafficking and could lead to a new approach to treat patients.

The post-translational modifications are important for the trafficking of Na^+^ channels (213). Specifically, the phosphorylation of Na^+^ channel protein process is well known to be a key factor in the trafficking (214, 215). The PKA, protein kinase C (PKC), and CAMKII are among the most abundant kinases expressed in the heart (216, 217). More recently, a study conducted on rabbit cardiomyocyte showed that the ubiquitination of Na^+^ channel by the ubiquitin ligase NEDD4 could regulate the endosomal trafficking (218). Targeting these enzymes could lead to enhancing the ion channel trafficking of Na^+^ channel and rescue the I_Na_ in HF. Interestingly, empagliflozin was recently described to reduce the I_NaL_ by inhibition of CAMKII activity (219). This medication could be used to restore a normal I_Na_ in patients with HF.

Changes in microtubule dynamics are known to alter ion channel trafficking. In fact, an upregulation of the detyrosination of microtubules can induce HF (Figure 3D) (187). The detyrosination is a post-translational modification of tubulin, the protein building block of microtubules, which are essential components of the cytoskeleton. In this process, the C-terminal tyrosine residue of tubulin is removed enzymatically, resulting in a stable and long-lived form of the microtubules. It was previously described that the detyrosination reduces the density and the peak I_Na_ in mice cardiomyocytes (220). It will be interesting to suppress the detyrosination to restore normal ion channel trafficking of the Na^+^ channel. Chen et al. showed that a pharmacological suppression of detyrosinated microtubules restores 50% of the lost contractile function in the left ventricular myocardium of a failing human heart (221).

Furthermore, Marchal et al. demonstrated in human-induced pluripotent stem cells-derived cardiomyocytes (hiPSC-CM) that the plus-end binding protein (EB1) improves the trafficking and the function of Na^+^ channel trafficking (190). In mammals, the EB protein family consists of three proteins: EB1, EB2, and EB3, which bind directly to the end of microtubules and facilitate the anchoring of proteins at the plasma membrane (222, 223). In addition, other microtubule plus-end tracking proteins, also called +TIP, have been found to be associated with the Na^+^ channel and potentially involved in its trafficking. For example, alterations in EB1, EB2, CLASP, CLIP1 CENP-F, MACF1, or iASPP are linked to a decrease in conduction or abnormal contraction, thereby emphasizing the impact of +TIP function on cardiac electrophysiology and function (Figure 3E) (189, 192, 224). One of the consequences of HF is the dysregulation of microtubule homeostasis, which is necessary for guiding ion channels to the membrane and this process explains the reduced I_Na_ reported in patients with HF.

### 4.2 Potassium current

Variants in K^+^ channels were the first described and represent the most important factor in the development of HF (52, 225). Indeed, a study described that 90% of *hERG* variants inhibited the trafficking from the ER to the Golgi (226). These variants cause *hERG* transport disorders by stimulating the UPR and leading the degradation of the ion channel protein (182). Variants on *KCNQ1* were also revealed to play a role in the abnormal *KCNQ1* trafficking, leading to HF and long QT syndrome (227, 228). Furthermore, another study described that a variant of *KCNQ1* impaired the *hERG* trafficking (229). Indeed, this variant of *KCNQ1* interacts in the cytosol with *hERG* and impairs the trafficking. The alteration of *hERG* trafficking, caused by the *KCNQ1* variant, leads to HF and long QT syndrome.

Hypokalemia or hyperkaliemia disturbs the trafficking of *hERG* (198). Guo et al. were the first to reveal how the density of *hERG* channels is regulated under hypokalemic conditions (230). This situation triggered an ubiquitination of *hERG*, internalization, and finally degradation (231) (Figure 3F).

The trafficking of K^+^ channels, especially *hERG* protein, involves a chaperone protein from the family of heat shock protein (HSP) (232, 233). HSP90 and HSP70 interact with *hERG* and play a crucial role for the maturation. In HF, the role of these chaperone proteins was not well described, but it was suggested as playing a potential role for therapeutic modulation of HSP in HF (196). Indeed, an inhibition of HSP90 in mice model of HF demonstrated a preserved left ventricular pressure (234). More recently, this team showed that simvastatin also inhibits HSP90 and reduces cardiac remodeling in HF rat model (235). For *hERG* protein trafficking, a study showed that the reduced expression level of *hERG* protein is linked with impaired trafficking because of the diminished interaction between *hERG* and HSP90 (Figure 3G) (197). This observation could explain the decrease of I_K_ observed in HF, and showed the potential therapeutic role of HSP90 in HF (236). There is limited knowledge regarding the trafficking defects for the I_to_ and I_K1_ in HF. However, the K_v_ channel-interacting protein (KChIP) is downregulated in HF and this contributes to the inhibition of the trafficking of I_to_ (237). Furthermore, one study mentioned the N-glycosylation defect of TREK channel, which contributes to reducing the I_K_ in HF (238).

### 4.3 Calcium handling

Ca^2+^ channel trafficking and function are regulated by auxiliary protein subunits, including the β-subunits, which help in their trafficking (239, 240). These β-subunits are important in facilitating the Ca^2+^ channel exiting from the ER (241), and different β-subunit variants have been involved in HF (180, 241). ER stress also induced a repression of Ca^2+^ channel trafficking. Indeed, a study conducted on hiPSC-CM showed that the activation IRE1, a pathway activated by the UPR response, decreases the protein level of the Ca^2+^ channel (Figure 3B) (186). In failing human hearts a decrease of Myc box-dependent-interacting protein 1 (BIN1) protein has been demonstrated. In myocytes, BIN1 facilitates microtubule-based delivery of Cav1.2 channels directly to T-tubules for normal Ca^2+^ transient development (242). BIN1 acts like a membrane scaffolding protein to anchor the Ca^2+^ channel at the plasma membrane and could explain the decrease of I_CaL_ observed in HF (193) (Figure 3E). In case of this channel, a recent study conducted in mice knock-out for CASK demonstrated that the loss of I_CaL_ accelerates HF development (194). This latest study demonstrated the importance of the actors involved in the trafficking of ion channels and that their destabilization can lead to aggravating the development of HF.

Furthermore, studies have shown that the post-translational modifications could affect the other components of the Ca^2+^ handling like the RYR2. PKA hyperphosphorylation, CAMKII, oxidation, and nitrosylation trigger the destabilization and dissociation of FKBP12.6 from the channel and contribute to SR Ca^2+^ leak that is thought to induce arrhythmias and decrease myocardium contractile strength in HF (243). These post-translational modifications can cause defects of RYR2 trafficking and contribute to development of HF.

Few studies attempted to understand the mechanisms of NCX trafficking in HF. In fact, the NCX activity in HF occurs more in response to a persistent I_Na_ that increases its reverse mode or by a Ca^2+^ leak coming from the SR, thus increasing its activity and its arrhythmic role. However, a recent study highlighted the role of the synapsin-2 on the NCX regulation of trafficking in HF (195). The synapsin-2 is a protein associated with the vesicle transport during protein trafficking. First, the authors showed a decrease of synapsin-2 expression in the left ventricle in mice model of HF and demonstrated a co-localization of synapsin-2 and NCX. These data suggest that reduced synapsin-2 in HF alters the NCX trafficking and leads to ventricular arrhythmias (Figure 3H) (195).

### 4.4 Connexin

For the Cx43, the localization of this hemichannel is at the intercalated discs. The trafficking of Cx43 involves a major plus-end EB1 protein (244, 245). EB1 allows anterograde movement to the intercalated discs by linking the microtubules to the desmosomes via the interaction with another plus-end protein (p150) (246). In case of HF, oxidative stress causes the microtubule plus-end EB1 dissociation from the end of the microtubules (Figure 3E). Consequently, it impairs the microtubule attachment to adherent junction structures and the delivery of Cx43 to the plasma membrane at the intercalated discs (191, 247). This observation could explain the decrease of Cx43 observed in patients with HF (248).

Collectively, ion channel trafficking disturbances lead to HF. A better understanding of these mechanisms allows to develop new therapeutic strategies or to better understand certain therapies used. First, targeting HSP chaperone proteins, suppressing microtubule detyrosination, or even targeting Rab recycling proteins could be interesting new strategies to restore ion channels trafficking. Therapy using an adeno-associated virus (AAV), as in the CUPID2 study, does not seem to be the best solution as of yet in the treatment of HF (111). However, therapies carried by SGLT2 inhibitors seem the most promising for trafficking restoration, specifically empagliflozin, which makes it possible to restore normal ion channels trafficking through its chaperone-like effect by increasing the forward trafficking of ion channels (249). Finally, Entresto® is a new therapeutic option to reduce the occurrence of arrhythmias in patients with HF. Its precise role in the treatment of these arrhythmias is still being explored, but it appears that its role affects the three pathways of B-type natriuretic peptide, angiotensin II, and bradykinin (250). Its role in ion channel trafficking is yet to be discovered.

## 5 Summary

Ion channel trafficking is a central regulator of cardiac electrophysiology, contractility, and alterations in the cell homeostasis in HF, thereby contributing to arrhythmias. Several actors are involved in this process, from the formation of new synthesized proteins to their assembly at the membrane. HF can induce an intracellular stress, which also disturbs the ion channels trafficking and accelerates the development of the electrical activity and function of the failing heart. However, few studies demonstrate the direct link between the HF and protein trafficking. A better understanding of the mechanisms leading to ion channels trafficking disorders in HF could open new therapeutic targets.
